# The Physician’s Visiting List

**Published:** 1887-01

**Authors:** 


THE PHYSICIANS' VISITING LIST. Thirty-sixth year, Philadelphia, Pa. Blakiston, Son & Co. 1887.

This visiting list is well bound in good leather, conveniently arranged and is compact, three essential qualities necessary for a good visiting list.

Its contents are arranged in the most advantageous way, including a calendar, table of signs, list of poisons and antidotes, dose tables, re-written in accordance with the sixth revision of the U. S. Pharmacopœia, Marshall Hall's ready method in asphyxia, lists of new remedies, Sylvester's method for producing artificial respiration, with illustrations, diagram for diagnosing diseases of the heart and lungs, explanation of the metric system, explaining the difference between it and the present system of weights and measures, a new table for calculating the period of utero-gestation and a number of other new features.

